# Accurate, precise modeling of cell proliferation kinetics from time-lapse imaging and automated image analysis of agar yeast culture arrays

**DOI:** 10.1186/1752-0509-1-3

**Published:** 2007-01-08

**Authors:** Najaf A Shah, Richard J Laws, Bradley Wardman, Lue Ping Zhao, John L Hartman

**Affiliations:** 1 Department of Computer and Information Sciences, University of Alabama at Birmingham, Alabama, 35294, USA; 2 Division of Public Health Sciences, Fred Hutchinson Cancer Research Center, Seattle, Washington, 98109, USA; 3 Department of Genetics, University of Alabama School of Medicine, Birmingham, Alabama, 35294, USA

## Abstract

**Background:**

Genome-wide mutant strain collections have increased demand for high throughput cellular phenotyping (HTCP). For example, investigators use HTCP to investigate interactions between gene deletion mutations and additional chemical or genetic perturbations by assessing differences in cell proliferation among the collection of 5000 *S. cerevisiae *gene deletion strains. Such studies have thus far been predominantly qualitative, using agar cell arrays to subjectively score growth differences. Quantitative systems level analysis of gene interactions would be enabled by more precise HTCP methods, such as kinetic analysis of cell proliferation in liquid culture by optical density. However, requirements for processing liquid cultures make them relatively cumbersome and low throughput compared to agar. To improve HTCP performance and advance capabilities for quantifying interactions, YeastXtract software was developed for automated analysis of cell array images.

**Results:**

YeastXtract software was developed for kinetic growth curve analysis of spotted agar cultures. The accuracy and precision for image analysis of agar culture arrays was comparable to OD measurements of liquid cultures. Using YeastXtract, image intensity vs. biomass of spot cultures was linearly correlated over two orders of magnitude. Thus cell proliferation could be measured over about seven generations, including four to five generations of relatively constant exponential phase growth. Spot area normalization reduced the variation in measurements of total growth efficiency. A growth model, based on the logistic function, increased precision and accuracy of maximum specific rate measurements, compared to empirical methods. The logistic function model was also more robust against data sparseness, meaning that less data was required to obtain accurate, precise, quantitative growth phenotypes.

**Conclusion:**

Microbial cultures spotted onto agar media are widely used for genotype-phenotype analysis, however quantitative HTCP methods capable of measuring kinetic growth rates have not been available previously. YeastXtract provides objective, automated, quantitative, image analysis of agar cell culture arrays. Fitting the resulting data to a logistic equation-based growth model yields robust, accurate growth rate information. These methods allow the incorporation of imaging and automated image analysis of cell arrays, grown on solid agar media, into HTCP-driven experimental approaches, such as global, quantitative analysis of gene interaction networks.

## Background

Most genetic research is aimed ultimately at understanding how phenotypes are produced. This is complicated by the fact that genes interact with the environment and other genes in producing phenotypes, such that the phenotypic effect of mutating any single gene depends on the allele status at secondary loci as well as environmental variables [1]. Large-scale phenotypic analysis of combinations of genetic and environmental variations (perturbations) has proven useful for understanding the organization of gene networks [2-4]. However, analysis of gene interactions is not tractable in humans due to their outbred nature and phenotypic complexity [5], thus genetically tractable model systems can provide new inroads for understanding genotype-phenotype complexity of human disease pathways [6,7]. In this regard, the collection of 5000 yeast gene deletion strains provides a unique resource for systematic analysis of gene interactions by comparing cell proliferation phenotypes (CPPs) of the WT strain and each deletion mutant under various perturbation conditions [2-4,8,9].

Most large-scale phenotypic analyses of the yeast gene deletion strains have been non-or semi-quantitative, based on end-point analysis of cell proliferation [10]. On a smaller scale, quantitative analysis of gene interactions has proven advantageous by virtue of being more objective, sensitive, and discriminating between strength of interactions, which can aid identification of distinct pathways represented within large sets of interacting genes [2,11-14]. Precise quantitative phenotyping together with kinetic analysis of cell proliferation can reveal differential genetic regulation of distinct physiological phases of growth [15,16]. Ideally, HTCP would have sufficient throughput *and *quantitative accuracy for investigating genotype-phenotype complexity with respect to many dimensions including time, different kinetic features of cell proliferation, gene-gene and gene-environment perturbation combinations, and gradients of perturbation intensity. These dimensions may be critical to parse gene networks functionally.

Turbidity readings of liquid cultures are the current standard for kinetic analysis of microbial cell proliferation [12,16]. However, throughput is greatly reduced, relative to endpoint analysis of agar spotted arrays, or the use of DNA microarray hybridization methods [4,8,17-19]. Throughput is lower for kinetic vs. endpoint analysis because ~30 time points of data are taken for each culture. Furthermore, liquid arrays are more difficult to analyze than solid arrays due to shaking requirements for resuspending cells prior to each reading, and increased time for operation of a microplate reader vs. visual inspection. Precision of kinetic turbidity readings is limited by spilling, cross contamination, and evaporation, which hinders miniaturization and automation of liquid culture-based HTCP. Phenotypic Array Analysis (PAA), an alternative quantitative HTCP approach based on time-lapse imaging and image analysis of agar spotted cell arrays, improves throughput to ~25,000–100,000 measurements per hour [2], taking advantage of the easy handling and potential for rapid imaging of agar cell arrays. This work describes YeastXtract, an image analysis software application that improves PAA, so that early phase kinetic growth rates can be measured, analogous to OD readings of liquid cultures. Validation experiments are presented for YeastXtract. Additionally, the logistic growth equation was used for kinetic modeling of cell proliferation data and shown to offer advantages over empirical growth models for quantifying cell proliferation phenotypes from time series images. Together, these methods are intended to improve HTCP capacity for global, quantitative analysis of gene interactions using large microbial mutant collections.

## Results and Discussion

### YeastXtract image analysis software

YeastXtract is a software application that analyzes time series images of yeast cell arrays, for the purpose of kinetic growth curve analysis, and can be used on operating systems with the Java platform installed. From the YeastXtract user interface, a sequence of images is selected using a 'Browse' function, and automated analysis is initiated by selecting the "Start Analysis" button. After analysis is complete, the enumerated intensities and areas of culture spots are displayed. Time-lapse images of individual spot cultures, along with plotted growth curves can be accessed via the 'Spot Level Information' tab. Accuracy of spot detection can be checked using the 'Spot Detection' function which depicts the ellipses used to quantify biomass of each culture on the cell array image. A user manual with screenshots depicting how these functions are accessed from the user interface is provided as [see Additional file 1]. The software executables, source code, and sample images are available for download [20] and use under the Creative Commons Attribution-NonCommercial-ShareAlike 2.5 license [21]. The software has a modular design to facilitate modification and further development. An overview of the analysis algorithm is provided below, with a detailed description in Methods.

**Figure 1:**
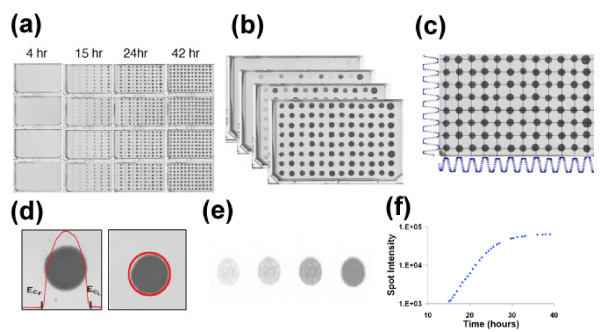
**An overview of the YeastXtract image analysis algorithm**. **(a) **A limited time series of four replicate cell arrays is shown. The arrays were created from serial 2-fold dilution of a 1:4 dilution of an overnight culture, skipping rows with each dilution and backfilling skipped rows. **(b) **A time series of one cell array from Panel A is shown at larger magnification. **(c) **Depiction of the first step of spot detection. A grid is created from the local maximum values after summing row and column pixel intensities over the entire array image (see Materials and Methods). The summed row and column pixel intensities are plotted at the edges of the array. **(d) **Depiction of the second step of spot detection. A cell containing each spot is defined by the 50 × 50 Pixel square surrounding the grid intersections shown in panel C. Within each cell, the horizontal and vertical diameters of each spot are calculated as the pixel distance between threshold values of summed column and row pixel intensities. An ellipse is drawn around each spot based on the resulting diameters. Spot detection is more precise for darker spots. Hence, the last time point is used for spot detection and those ellipses are used for extracting signal intensities from aligned time series of images of each cell array. **(e) **After spot detection, the background local to each spot is subtracted and remaining signal intensities are calculated by summing pixel values inside each ellipse. **(f) **Spot intensities are plotted versus time and used for growth modeling.

Up to ten cell arrays are imaged at a time, using an optical scanner (Fig. 1a), as previously described [2]. Each cell array time series is analyzed individually (Fig. 1b). Images from a time series are aligned in reverse-chronological order using a least squares algorithm. The final time point is used for spot detection (Figs. 1c,1d), and the resulting 'grid' is used for spot extraction from aligned images (Fig. 1e). Spot detection is performed in two steps. First, the approximate center of each spot is determined from local maxima of summed pixel columns and rows (Fig. 1c). Second, the pixel columns and rows in each cell of the resulting grid are analyzed to identify the horizontal and vertical diameters of each spot, from which an ellipse is calculated (Fig. 1d). For signal extraction, the background of each spot is computed from a localized mean around the mode of pixel intensities outside the ellipse, and then subtracted from intensities of each pixel inside the ellipse (Fig. 1e). Total pixel intensity is calculated for each time-point, and the intensities are plotted vs. time (Fig. 1f). The pixel area of each ellipse is calculated in order to normalize spot intensities against spot size.

### YeastXtract provides accuracy and precision for image analysis of agar culture arrays comparable to optical density readings of liquid cultures

The original aim of this study was to increase the sensitivity for detecting spotted cell cultures to reach the range and accuracy of microplate readers for kinetic growth analysis. Our previous image analysis programs did not have the sensitivity to measure specific growth rates when they were in their maximal steady state [2]. Spot detection and local background subtraction were implemented to increase accuracy and precision of intensity measures. Background subtraction is also useful for modeling growth phenomena, since the background is non-biological and can contribute substantially (~25%) to the final spot intensity.

**Figure 2:**
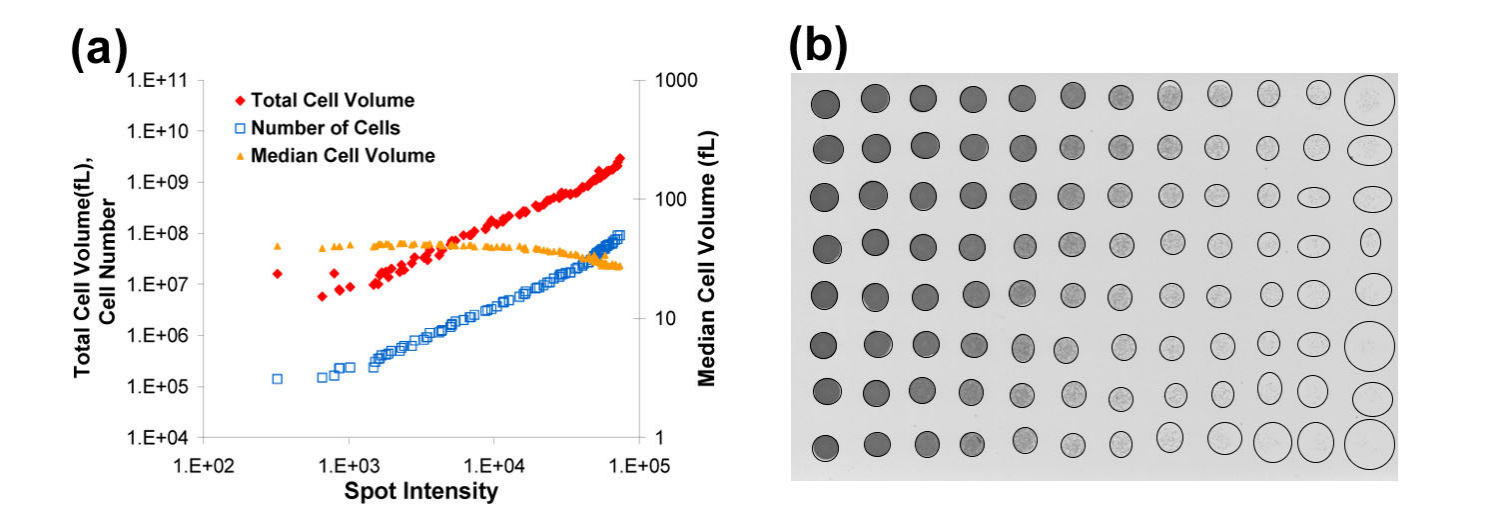
**Biomass correlates linearly with spot intensity of imaged cultures**. An overnight culture was first diluted 1:4 in water and then serially diluted (3:4) in column A of a 96 well plate. All cultures were then 2-fold serial-diluted across each row, and 4 μL of the resulting cell suspension was spotted to agar media. The cell array was imaged 23 hours after spotting and cultures were immediately excised and resuspended in 2 mL of ice-cold water. Biomass was calculated by particle size analysis of each culture resuspension. The data used to generate this figure are in Additional file 2. **(a) **The numerical output (spot intensity) from YeastXtract is plotted vs. biomass for each culture. Biomass is calculated as the total cell number times the median cell volume (also plotted for each culture). **(b) **The image used for this analysis is shown along with the ellipses used for signal extraction.

Particle size analysis (Z2 Coulter Counter, Beckman) was used to determine the correlation between biomass (total cell volume) of a spot and its spot intensity measurement (Fig. 2a, see Additional file 2). A gradient of dilutions (from 1:4 to 1:60,000) of an overnight culture were spotted onto a 96-culture array. After 23 hours, the array was imaged (Fig. 2b) and all cultures were immediately excised and subjected to particle size analysis. After image analysis, spot intensities were plotted vs. total cell volume (Fig. 2). The densest culture spots had intensity of about 7.5 × 10^4 ^pixels (spot area of ~610), and contained approximately 9 × 10^7 ^cells having a total cellular volume of ~3 × 10^9 ^fL. Including average pixel intensity background of ~37, the average spot culture pixel intensity was approximately 158. Thus, given the pixel intensity of 8-bit images ranges from 0 to 255, final spot intensities reach only ~65% image saturation. Linear regression of image intensity vs. biomass (total cell volume) of spot cultures revealed a high degree of correlation (R^2 ^= .94). Total cell volume had slightly higher linear correlation than cell number (R^2 ^= .92), due to a slight reduction in median cell size as cultures approached their final population density (Fig. 2a). It can be concluded from Fig. 2 that PAA-derived spot intensities are comparable to OD measurements of liquid cultures, with respect to accuracy and precision for quantifying cell proliferation.

**Figure 3:**
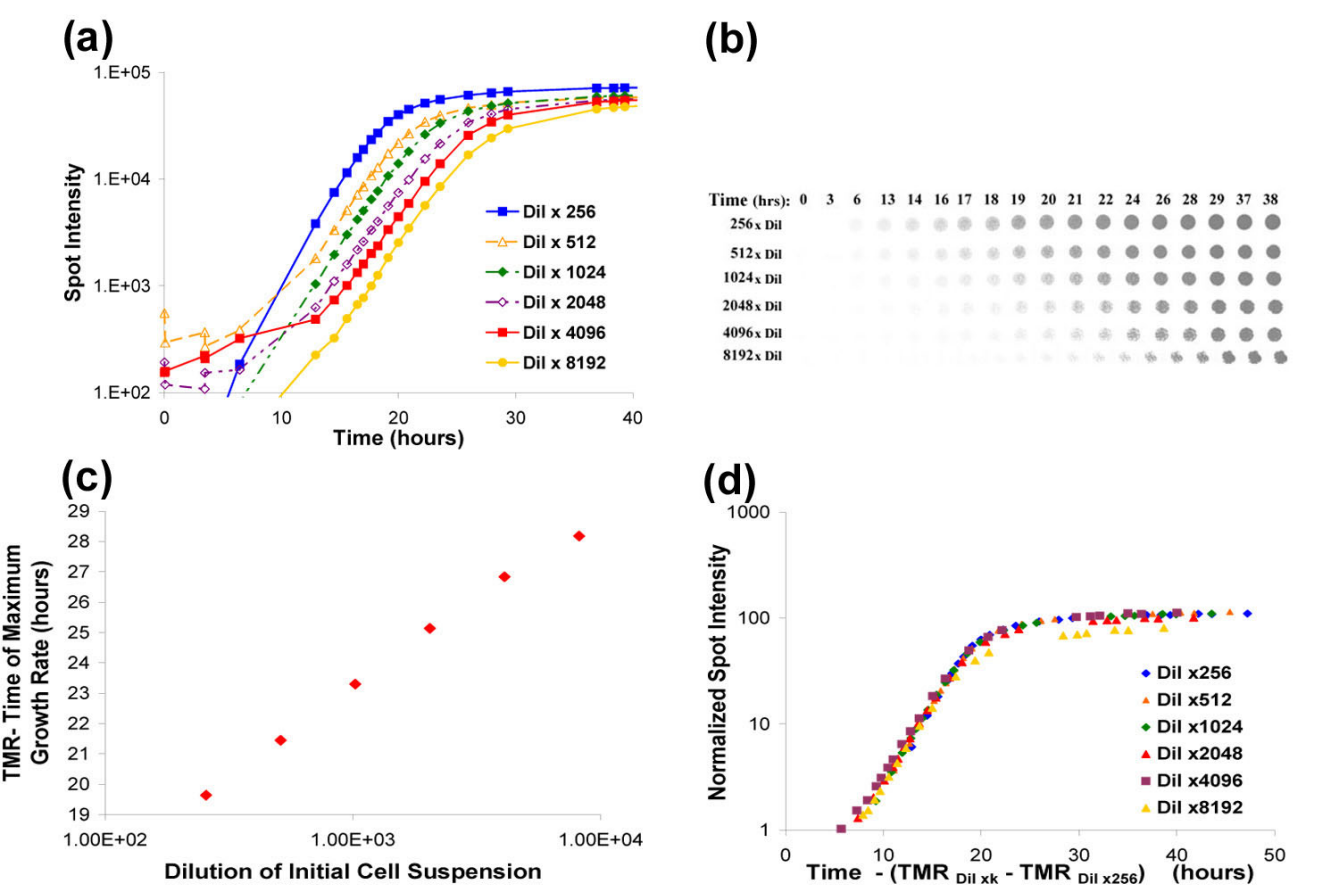
**Spot intensities of culture images are used for kinetic analysis of proliferation**. An overnight culture was serially diluted, by 2-fold, across a 96- well plate. **(a) **Raw image intensities are plotted versus time for a representative culture at each dilution. **(b) **The images of spot cultures for each data point in panel (a) are shown. **(c) **After fitting the data to the logistic equation, TMR, which is the time at which the overall population growth rate is maximal, was calculated for each curve and is plotted versus time. The difference between values of TMR for each curve reflects the doubling time, since cultures were created by serial two-fold dilution. **(d) **The spot intensities were normalized by spot area, and curves were shifted on the time axis by the difference between TMR of the culture and TMR of the 256x-diluted culture.

Four microliters of culture suspension is typically used for spotting cultures, giving rise to a spot area of approximately 625 pixels (25 × 25) on a 600 × 400 pixel array (140 dpi resolution image of standard SBS microplate). Spot cultures are detected when the average pixel intensity is approximately one (Fig. 3d). A constant exponential rate of growth is observed over 4–5 generation times (Figs. 3a and 3d). The final population intensity (FPI), reflecting total growth efficiency when resources for cell proliferation are exhausted, is typically (normalized by spot area) around 100–120. TMR (time when maximum growth rate is observed) is the time it takes a culture to reach its maximum growth rate (see kinetic growth modeling in Methods). Thus, the difference in TMR between two-fold dilutions of a culture approximates the minimum doubling time (Fig. 3c). Shifting 2-fold diluted cultures by TMR yields overlapping growth curves (Fig. 3d).

### Normalization of spot intensity by spot area reduces variation in FPI and AUGC

**Figure 4:**
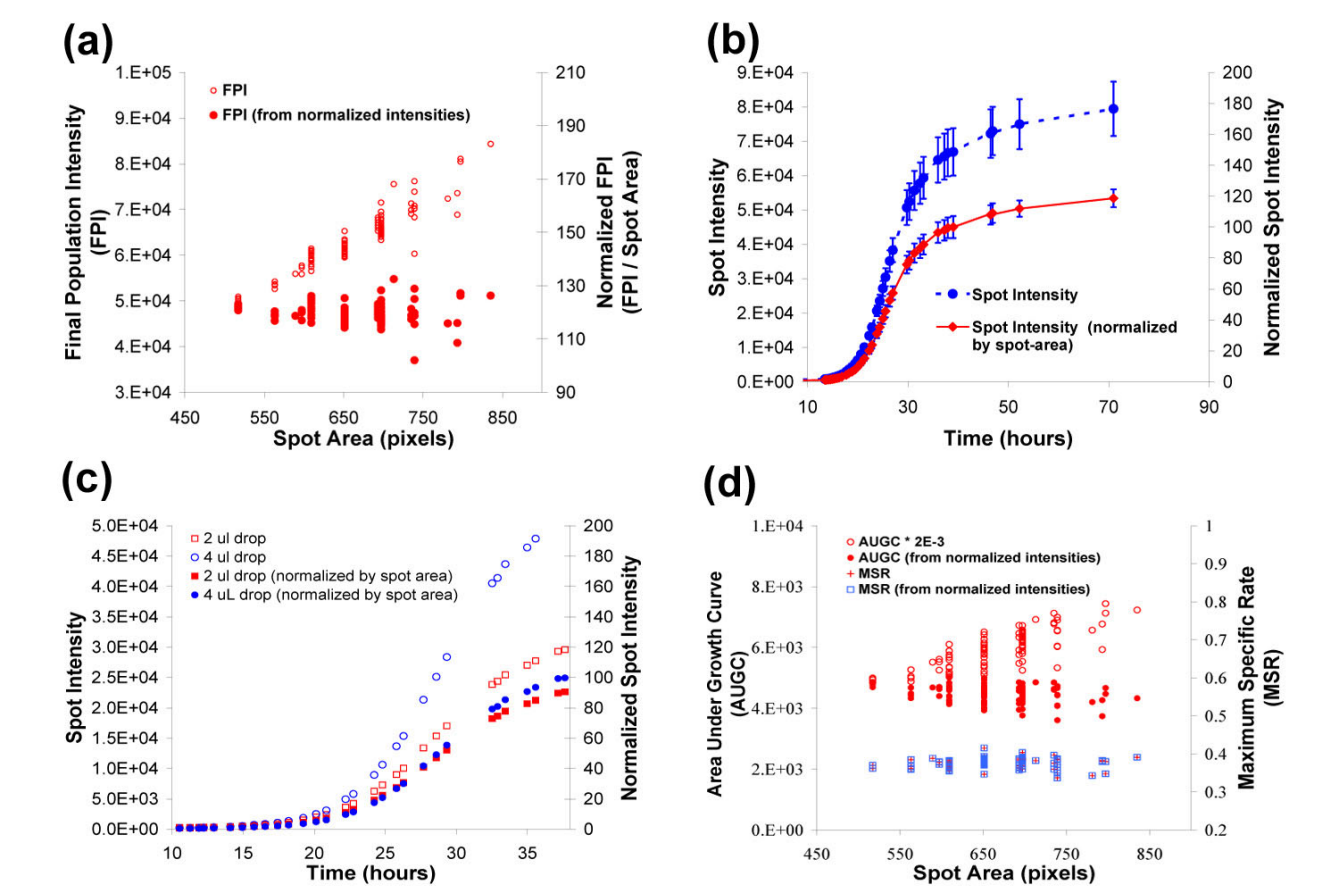
**Normalizing spot intensity by spot area increases precision of growth curve analysis**. An overnight culture was diluted 1:2000 and distributed into a 96 well plate. Agar arrays were printed using 2 uL and 4 uL drops. A time series of images was collected for 72 hrs. The data used to generate this figure are in Additional file 2. **(a) **From the final time point, spot intensity is plotted against spot area (scale to left), and normalized spot area is also plotted (scale to right). **(b) **Averaged data from all 96 cultures (4 μL drop array) are plotted for normalized and non-normalized spot intensities. Standard deviation bars show the effect of spot area normalization on measurement variation across time. **(c) **To further see the effect of normalization, arrays made with 2 μL and 4 μL spots from the same starting culture were analyzed. The averaged data from each set of 96 cultures, normalized and non-normalized, were plotted vs. time. **(d) **To observe the effect of spot area normalization on the MSR and AUGC, non-normalized and normalized values for each CPP were plotted vs. spot area.

An important difference between liquid and agar culture analysis is that the area of the culture spot affects the reading (Fig. 4a). Hence, normalizing spot intensity data by spot area can reduce experimental noise, since spot area variation is mostly non-biological (Fig. 4b). The utility of spot area normalization was tested by intentionally varying the spot size, and normalization was found to correct almost entirely for the effect of spot size on growth curve differences (Fig. 4c, see Additional file 3). FPI reflects the carrying capacity (total growth yield, or efficiency) of a culture [22,23]. Since there is variation in the areas of cultures even when equal volumes are used to print each spot, spot area normalization is needed to accurately compare growth efficiency. In summary, spot area normalization reduces variation in FPI (final population intensity) and AUGC (area under growth curve), while not affecting MSR (maximum specific rate) or TMR calculations (Fig. 4, Tables 1 and 2).

### A logistic function model is used to quantify cell proliferation phenotypes, such as maximum specific rate and total growth efficiency, from time series data

**Figure 5:**
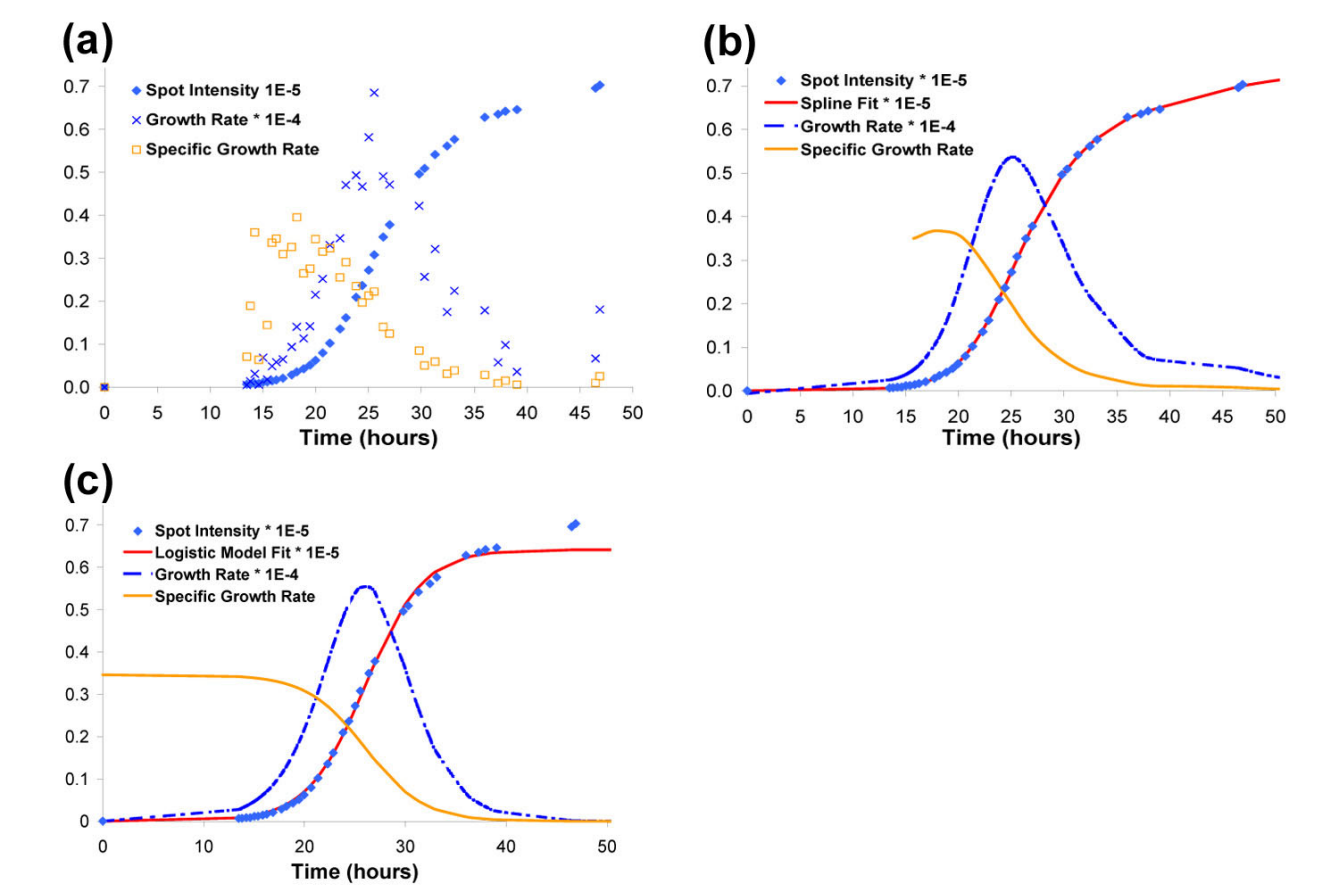
**Spot intensity time series data are accurately modeled by the logistic growth equation**. Spot intensity data from a typical spot culture, on the edge of a cell array, were used to illustrate three different growth models. **(a) **Raw spot intensity is plotted versus time. Also plotted are the growth rate and specific growth rate, as calculated directly from the raw data. **(b) **A spline was used to fit the raw data from panel A. The raw data are plotted vs. time, along with the fitted growth curve, growth rate, and specific growth rate. **(c) **The logistic growth equation was used to fit the raw data and to calculate growth rate and specific growth rate. Refer to Tables 1 and 2 for comparison of cell proliferation phenotype values obtained by each model.

Different attributes of growth curves represent distinct physiological phases of growth [16]. When a fresh culture is inoculated from a saturated, stationary culture, there is typically a 'lag' phase until the culture doubling time reaches a minimum. The population then undergoes a phase of growth during which the overall growth rate increases exponentially while the specific rate, or percent change in population with respect to time, remains constant (Fig. 5). Finally, when resources supporting growth become limiting, the growth rate decays until growth ceases and the "carrying capacity" is thus reached. These physiologically distinct characteristics of growth are potentially under the control of different genes and pathways and can thus be considered as different cell proliferation phenotypes (CPPs). In this study, we focused on the following CPPs:

• Total Growth Efficiency, which is measured by the Final Population Intensity (FPI) of a spot culture, is also referred to as the carrying capacity in the logistic equation.

• Specific Growth Rate is the growth rate divided by the population size.

• Maximum Specific Growth Rate (MSR) is the maximum value of the specific rate over time, and is inversely proportional to the minimum doubling time of a culture.

• Doubling Time is the time required for the population size to double. Minimum doubling time is equal to log_e _2/MSR.

• Area Under Growth Curve (AUGC) is the integral of spot intensity curve over the interval between the first and final time point.

• Time of Maximum Rate (TMR) corresponds to the time when the growth rate reaches its peak value; by the logistic model, TMR marks the time when half carrying capacity is reached.

• Lag Time is a property of the culture, whereby there is a delay after cells are introduced into a new medium before MSR is achieved.

To evaluate the performance of different growth models, we considered reduction in the variation of CPP values from many replicate cultures as an increase in the precision of a model (Tables 1 and 2). The following form of the logistic equation was used to fit growth data:

G(t)=K1+e−r(t−l),G(0)<K
 MathType@MTEF@5@5@+=feaafiart1ev1aaatCvAUfKttLearuWrP9MDH5MBPbIqV92AaeXatLxBI9gBaebbnrfifHhDYfgasaacH8akY=wiFfYdH8Gipec8Eeeu0xXdbba9frFj0=OqFfea0dXdd9vqai=hGuQ8kuc9pgc9s8qqaq=dirpe0xb9q8qiLsFr0=vr0=vr0dc8meaabaqaciaacaGaaeqabaqabeGadaaakeaacqWGhbWrcqGGOaakcqWG0baDcqGGPaqkcqGH9aqpdaWcaaqaaiabdUealbqaaiabigdaXiabgUcaRiabdwgaLnaaCaaaleqabaGaeyOeI0IaemOCaiNaeiikaGIaemiDaqNaeyOeI0IaemiBaWMaeiykaKcaaaaakiabcYcaSiabdEeahjabcIcaOiabicdaWiabcMcaPiabgYda8iabdUealbaa@44FC@

where K ("carrying capacity") is approximated by the FPI; *r *is the MSR, and *l *is the TMR. We compared CPPs derived from the logistic equation model, the raw data, and data fit to a spline model (see Methods for more details about the models).

### The logistic function growth model increases precision of MSR and TMR measurements

Median MSR values were comparable, regardless of the model used for calculation (Table 1), with minimum doubling times ranging between 1.75 (MSR = .40) and 1.98 (MSR = .35) hours. However, the variation in MSR values was reduced by 63% (24% vs. 9%) if calculated using spline-fit data instead of raw data (Table 1, see Additional file 4). MSR variation was reduced another 44% (9% vs. 5%) using the logistic model (Table 1). Variation in the calculation of TMR was similarly improved by the spline- and logistic equation-fitted data. The likely explanation for the reduced variation in the spline-fit vs. the raw data is that growth is a continuous function, and thus fitting of the data increases precision by reducing the time interval for rate calculations. Increases in measurement precision for MSR and TMR with the logistic equation may stem from it being specifically designed for modeling growth phenomena [22,23].

AUGC measurements were not greatly impacted by the model used. Likewise, FPI, which is a dominant factor in AUGC calculation, is relatively unaffected by model selection (Table 1). There was a trend toward lower FPI and AUGC with the logistic model (Fig. 5), which was investigated by examining the nature of FPI in more detail, as described below.

### An 'initial carrying capacity' is modeled by the logistic equation

**Figure 6:**
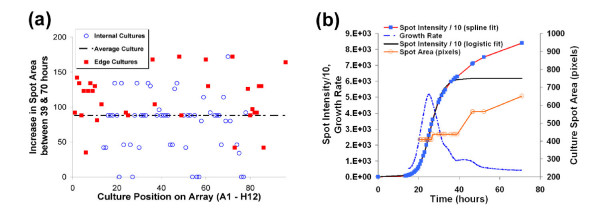
**Data filtering is used to reduce the variable effect of spot area increases on growth curve modeling**. **(a) **The increase in spot culture area, between 39 and 72 hours, is plotted for 96 replicate cultures (an 8 × 12 cell array). Internal and edge cultures are labeled differently to highlight the increases in spot area of edge cultures. **(b) **The spot intensity, spot area, growth rate (derived from a spline fit), and logistic-fitted growth curve are plotted vs. time to illustrate that the initial carrying capacity is reached about the time that the spot area begins to increase (after ~40 hrs in this example). Hence, late data are filtered to avoid the effect of this artifact on growth modeling with the logistic equation (see Materials and Methods).

The trend toward lower FPI and AUGC with the logistic model (Tables 1 and 2) was caused by underestimation of spot intensity at later times, particularly for cultures spotted on the edge of an array (Figs. 5 and 6). It was frequently observed, in images from late time points, that spots around the edges of the array tend to have larger areas than interior-located spots. Thus, we hypothesized that increases in spot intensity, if due to increases in spot area at late time points, would not be well modeled by the logistic equation. As the hypothesis goes, once the spot has grown to confluence, it reaches an 'initial carrying capacity', however due to a residuum of energy sources, cultures can continue to grow slowly (with non-logistic kinetics). Since the cultures have grown to confluence, new cells begin to become outwardly displaced, resulting in an increasing spot area. Since the cultures on the edge of the array have less competition for available nutrients, the spot areas can increase more.

In Fig. 5, these phenomena are depicted by a time series of spot intensities for a typical edge culture, where an inflection in the growth curve occurs after initial carrying capacity is reached (between 40 and 45 hrs). Fitting the data to a spline, the late increase in spot intensity is followed closely (Fig. 5b). However fitting the same data to the logistic equation, this inflection in the spot intensity curve is missed (Fig. 5c). In summary, the area of agar initially covered by cells at the time of array printing grows to confluence, reaching an "initial carrying capacity"; and further increases in spot intensities are correlated with actual increase in the size of the spot (Fig. 6), which is not well modeled by the logistic equation.

### Data are filtered after the time initial carrying capacity is reached to improve modeling

To better understand the nature of the initial carrying capacity, the difference in spot area after 39 and 70 hours of growth was examined, confirming that edge cultures increase in size more than internal cultures (Fig. 6a). We next examined the growth rate with respect to time and spot area, finding that increases in spot area correspond with an inflection in the growth rate curve (Fig. 6b). Thus, once spot cultures have reached their initial carrying capacity (the maximum population yield over the original area for the spotted culture), further increases are associated with increases in the spot area, occurring preferentially at the edges of a cell array.

To improve growth curve modeling with the logistic equation, we designed a filtering algorithm to reduce the effects that increases is spot area might have after initial carrying capacity is reached, since individual cultures in an experiment might have varying growth rates due to gene deletions and/or other perturbations. Since the logistic equation has the property that the maximum growth rate occurs when population is at half of carrying capacity, we used a spline to estimate the TMR and then filtered out time points having greater than 2.2 times the spot intensity at TMR. The filtering algorithm improves fitting of data to the logistic model by reducing the tendency for artificial increases in FPI for cultures on the edge of an array (Fig. 6b).

### Physiological lag time can be measured directly by Phenotypic Array Analysis

**Figure 7:**
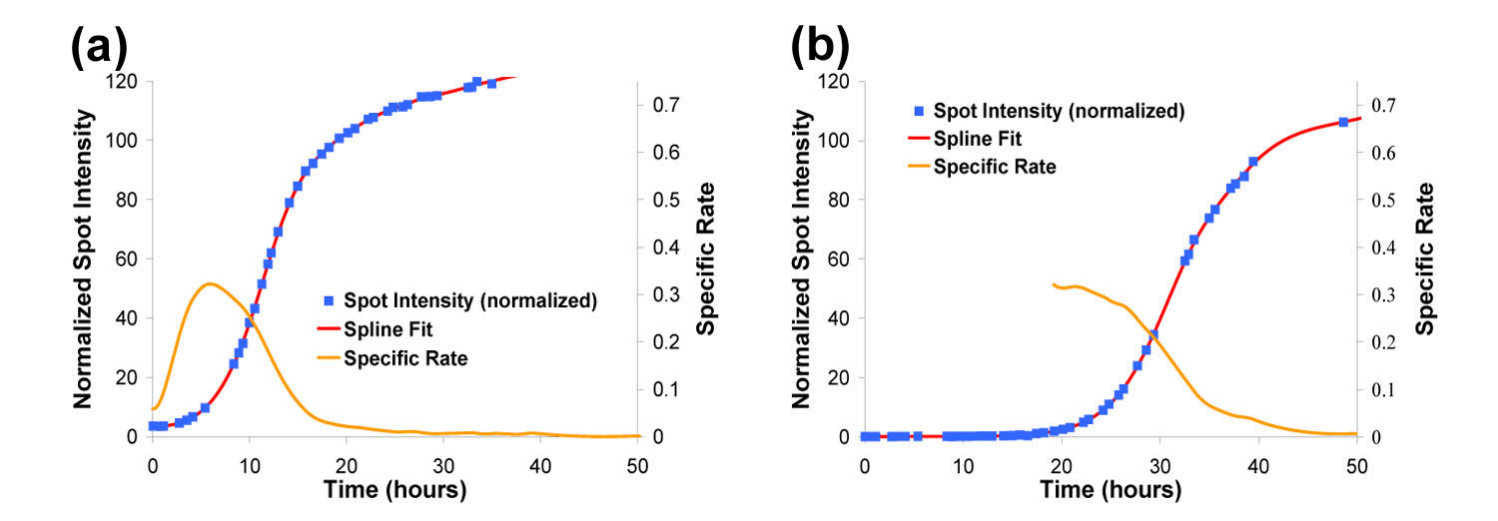
**Dense initial population cultures can be used to measure lag time**. Lag time is defined as the delay that a culture demonstrates from the time it is freshly inoculated to the time that it achieves its minimal doubling time. The spline model was used to measure lag for a representative culture printed at **(a) **lower dilution (1:4) and **(b) **higher dilution (1:2000). In panel (a), lag time can be directly observed, because the spot is detectable (average pixel intensity > 1) at time = 0. In contrast, in panel (b) cultures have passed through lag phase and exhibit MSR by the time spot intensities reach the threshold of detection.

An assumption of the logistic equation is that the MSR occurs at time = 0 (Fig. 5c). However, realistically there is a physiological lag time that occurs when a culture having approached carrying capacity, is again inoculated into fresh media conditions. The lag time is typically 1–2 generation times, but of variable duration. Since, with PAA, growth is analyzed over nearly 20 generations, the effect of lag on the logistic model is negligible (Tables 1 and 2). However, since the lag time is of biological significance and interest, we investigated use of the spline model for directly measuring the lag time from cell array images (Fig. 7). For this experiment, the same 'overnight' starting culture was diluted either 4-fold or 2000-fold before printing to different arrays. The lag time (the time for a culture to reach MSR) was ~5 hours (Fig. 7a). The more highly diluted culture achieved the same MSR (~.32), which was observed at the time the spot intensity breached the threshold of image detection (Fig. 7b). Thus, lag time and MSR can be measured together by printing arrays with low-dilution cultures.

### The logistic equation-based growth model is robust against data sparseness

**Figure 8:**
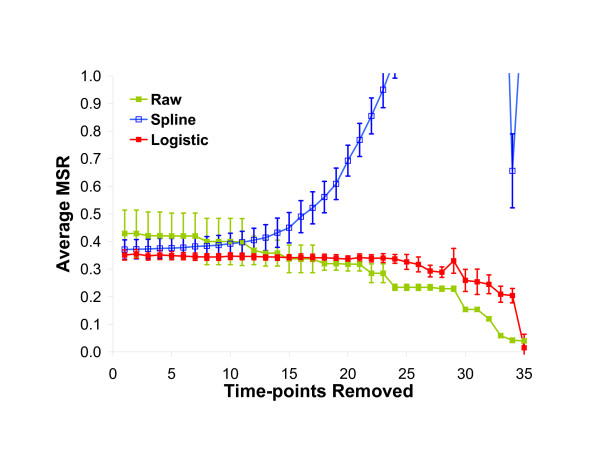
**The logistic growth equation model is relatively robust against sparse data**. Using the data represented in Tables 1 and 2, time points were randomly removed [see Additional file 5], and MSR was recalculated using each growth model. Average MSR (with standard error bars; n = 96) is plotted against the number of data points removed. The logistic model exhibits lower variation between replicates and is more precise as data is removed.

Once it was realized that the logistic equation was an accurate model for characterizing yeast cell proliferation, it became evident that it should be more robust than the spline or raw models to data sparseness because its parameters are more constrained. To assess model stability, individual time points were randomly removed one at a time (from a set of 38 time-points, collected over 70 hours), and MSR values were re-calculated from the remaining data (Fig. 8, see Additional file 5). The accuracy and precision of the average MSR value calculated from the logistic model was greater than that calculated by the spline model or using raw data (Fig. 8).

The robustness of the CPPs obtained from the logistic model likely results from the appropriateness of assumptions inherent to its equation for cell proliferation phenomena; the main assumption being that the rate of increase in biomass at any time is proportional to the biomass and the availability of resources [22,23]. A major strength of this form of the logistic equation is that its two major parameters, *K *and *r*, correlate well with FPI and MSR under standard conditions for growing spotted cultures on agar media.

## Conclusion

Global, systematic analysis of gene interaction networks is a recent experimental paradigm for systems biology. Since genetic interactions are often scored on the basis of cell proliferation measurements, HTCP is an enabling technology for this field of research. YeastXtract and the growth modeling algorithms presented here, help advance HTCP throughput and accuracy to enable phenotypic measurements in different dimensions such as varying intensities of perturbations, and different physiological aspects of growth responses (e.g., lag, maximum growth rates, and total growth efficiency). These advances will allow interactions to be investigated not only from the perspective of different combinations of gene and environmental/chemical perturbations, but also different aspects of the growth phenotype itself, each of which may be sculpted by different natural selective pressure for gene activities.

In a previous publication, we described Phenotypic Array Analysis, an HTCP method based on rapid imaging of ~25,000 spotted cultures per hour [2]. YeastXtract now enables automated PAA, without need for manual pre-processing of images. It provides single pixel resolution, improving PAA sensitivity and accuracy. While the methods were developed using yeast, and intended for application to the set of 5000 yeast gene deletion strains, they should also be applicable to other cell types that can be grown in similar fashion as agar cell arrays. Imaging and automated image analysis of cell arrays can now be incorporated into HTCP-driven experimental approaches, such as for quantitative investigations of gene interaction networks [1,2]. Looking forward, insight from global, quantitative analysis of gene interaction networks in single cell organisms, should be extensible for hypothesis-driven investigations of cellular pathways that buffer genetic and environmental perturbations in an orthologous fashion in multi-cellular organisms [24,25].

## Methods

### Strains and media

All experiments were performed with BY4741 strain (MATa *ura3 leu2 his3 met15*). Pre-growth was in YPD liquid media, dilutions were in water, and growth measurements were on synthetic complete media [26].

### Cell array printing and imaging

Cultures were grown as a single overnight culture and diluted in water prior to spotting 4 μL drops onto agar plates containing synthetic complete media, as previously described [2]. The plates were incubated at 30°C, and periodically removed and imaged on an Epson Expression 10,000 XL scanner operating in transmitted light mode. Images were collected at 140 dpi and 8-bit grayscale. Time stamps on the image files were used for generating growth curves after image analysis.

### YeastXtract (image analysis)

The algorithm was devised by building upon experience gained from development of a previous software program, SignalViewer [27,28], and consists of three main processes:

#### 1. Plate extraction and alignment

A set *S*, consisting of a time series of images of up to 10 cell arrays, was processed as a group. Thus, for a single scan configuration imaged *k *times, the image analysis algorithm requires the following input:

• *S*, a set of *k *TIFF images,

• *n*, the number of plates on the scan,

• *p*, the pitch, or the expected distance (in pixels) between the centers of two adjacent spots,

• *d*, the approximate expected length in pixels of a typical spot's diameter,

• *L*, a set of pre-defined horizontal and vertical coordinates that denote the location of each cell array ('plate') on a 'scan', containing up to 10 plates

• *r*, number of culture rows on each array, and

• *c*, number of culture columns on each array.

The pre-defined pixel coordinates in *L *for the position of each plate on a scan are used to extract each plate at all *k *time points. Because plates are manually placed on the scanning surface, a particular plate can be in slightly different locations on scans imaged at two different times. To minimize the effect of translocation on extraction of spot intensities, all *k *images are aligned using a least squares algorithm. Beginning with the next-to-last time-point, each image is aligned with the image immediately after it in time. Using the later image as a reference, the image is shifted by -α to +α pixels in the horizontal direction and -β to +β pixels in the vertical direction and the squared-difference in the pixel intensities of the two images is calculated for each combination of α and β. The image is shifted by the combination of α and β that results in the lowest difference between the two images. Using α = β = 4, the best alignment among 81 possible is selected.

#### 2. Spot detection

During the spot "detection" phase, the final image from the time series is used to identify the spot locations. First, the rectangular regions containing each spot are determined by considering columns of pixels one at a time. The 75^th ^percentile value of the pixel intensities in each column is calculated and the resultant value is stored in an array. This procedure is repeated for all pixel rows of the plate image and the intersection of the peak values of rows and columns having the highest 75^th ^percentile values are used to identify the approximate center of each spot, as depicted in Figure 1c. However, before detecting the peaks, the values in the row and column percentile arrays are processed using the LOESS smoothing algorithm with a smoothing parameter value of 0.03; we have found that this additional processing makes the algorithm more robust by filtering away noise on the image that may cause the algorithm to erroneously detect spot culture centers. The intersections of the row and column peaks form a grid representing the approximate locations of the spot centers. Given these centers and *p*, the approximate pitch, the rectangular region encapsulating a culture spot (approximately *p*^2 ^in size) can be extracted from the plate image. This procedure is repeated for all culture spots on the plate.

Next, the precise position of each spot within its region is determined by again identifying peaks in the row and column percentile arrays. All *k *images of each culture spot are collected and then aligned using the least squares method described for aligning whole plates. The image of the culture spot from the final time point is analyzed to determine the coordinates of an elliptical region that circumscribes the spot by summing the pixel intensities in each column and row. LOESS smoothing algorithm was used to process row and column sums with a smoothing parameter of 0.25. The locations of the peaks and the locations where the row and column sums rise above a threshold are used to compute the horizontal and vertical coordinates of the center and the two diameters of the ellipse, respectively (Fig. 1d):

• x2a2+y2b2=1
 MathType@MTEF@5@5@+=feaafiart1ev1aaatCvAUfKttLearuWrP9MDH5MBPbIqV92AaeXatLxBI9gBaebbnrfifHhDYfgasaacH8akY=wiFfYdH8Gipec8Eeeu0xXdbba9frFj0=OqFfea0dXdd9vqai=hGuQ8kuc9pgc9s8qqaq=dirpe0xb9q8qiLsFr0=vr0=vr0dc8meaabaqaciaacaGaaeqabaqabeGadaaakeaadaWcaaqaaiabdIha4naaCaaaleqabaGaeGOmaidaaaGcbaGaemyyae2aaWbaaSqabeaacqaIYaGmaaaaaOGaey4kaSYaaSaaaeaacqWG5bqEdaahaaWcbeqaaiabikdaYaaaaOqaaiabdkgaInaaCaaaleqabaGaeGOmaidaaaaakiabg2da9iabigdaXaaa@39D4@ (General equation of an ellipse)

• *E*_*cf *_first pixel column where column sum is greater than threshold,

• *E*_*cl *_last pixel column where column sum is greater than threshold,

• *E*_*rf *_first pixel row where row sum is greater than threshold,

• *E*_*rl *_last pixel row where row sum is greater than threshold,

• *a *= *E*_*cl *_- *E*_*cf*_

• *b *= *E*_*rl *_- *E*_*rf*_

• *h *= pixel column where column sum is highest

• *k *= pixel row where row sum is highest

#### 3. Signal extraction

The background of the culture spot is determined by computing the mode of the intensities of the pixels outside the ellipse, but within the area containing the ellipse, and then taking a local average around that mode. This background intensity is then subtracted from all images of this culture spot. For each image belonging to a particular culture spot, pixel intensities inside the ellipse are summed. The area of the ellipse circumscribing each culture spot is calculated by counting the number of pixels inside the ellipse.

### Spot culture biomass measurements

For figure 3, 96 spot cultures were cut out immediately after imaging and resuspended in 2 ml of ice-cold water by vortexing the agar plug. An appropriate fraction of the cell suspension was then taken for particle analysis (~5 × 10^6 ^total cells), and transferred to 10 mL of ice cold saline (Isoton, Beckman). A Z2 Coulter Counter (Beckman) with 70 um aperture (particle size 10 – 350 uL) was used for particle analysis.

### Kinetic growth modeling

Custom Matlab programs (available at [20]) were used for modeling growth curves from kinetic spot intensity data. Three different methods were used to calculate Cell Proliferation Phenotypes for 96 cultures from spot intensities. CPPs were calculated directly from the raw spot intensities in the first method and from logistic and spline-fitted models in the second and third methods, respectively. For the first method, the final recorded intensity was used as the FPI, Riemann sum was used to calculate the AUGC, and the MSR was determined by calculating the percent change in spot intensity with respect to time between consecutive points and recording the maximum among those values, as follows:

• ***G***_***raw***_(*t*) = Spot intensity at time *t*.

• **FPI**_***raw ***_= Spot intensity at final time-point; i.e. **G**_***raw***_(***t***_***final***_).

• AUGCraw=∑i=1nG(ti)∗Δti
 MathType@MTEF@5@5@+=feaafiart1ev1aaatCvAUfKttLearuWrP9MDH5MBPbIqV92AaeXatLxBI9gBaebbnrfifHhDYfgasaacH8akY=wiFfYdH8Gipec8Eeeu0xXdbba9frFj0=OqFfea0dXdd9vqai=hGuQ8kuc9pgc9s8qqaq=dirpe0xb9q8qiLsFr0=vr0=vr0dc8meaabaqaciaacaGaaeqabaqabeGadaaakeaaieqacqWFbbqqcqWFvbqvcqWFhbWrcqWFdbWqdaWgaaWcbaacbmGae4NCaiNae4xyaeMae43DaChabeaakiabg2da9maaqahabaGaem4raCKaeiikaGIaemiDaq3aaSbaaSqaaiabdMgaPbqabaGccqGGPaqkcqGHxiIkcqqHuoarcqWG0baDdaWgaaWcbaGaemyAaKgabeaaaeaacqWGPbqAcqGH9aqpcqaIXaqmaeaacqWGUbGBa0GaeyyeIuoaaaa@4877@ where *n *= number of time-points - 1 (Riemann sum).

• Rateraw(ti)=G(ti)−G(ti−1)ti−ti−1
 MathType@MTEF@5@5@+=feaafiart1ev1aaatCvAUfKttLearuWrP9MDH5MBPbIqV92AaeXatLxBI9gBaebbnrfifHhDYfgasaacH8akY=wiFfYdH8Gipec8Eeeu0xXdbba9frFj0=OqFfea0dXdd9vqai=hGuQ8kuc9pgc9s8qqaq=dirpe0xb9q8qiLsFr0=vr0=vr0dc8meaabaqaciaacaGaaeqabaqabeGadaaakeaaieqacqWFsbGucqWFHbqycqWF0baDcqWFLbqzdaWgaaWcbaacbmGae4NCaiNae4xyaeMae43DaChabeaakiabcIcaOiabdsha0naaBaaaleaacqWGPbqAaeqaaOGaeiykaKIaeyypa0ZaaSaaaeaacqGFhbWrcqGGOaakcqWG0baDdaWgaaWcbaGaemyAaKgabeaakiabcMcaPiabgkHiTiab+DeahjabcIcaOiabdsha0naaBaaaleaacqWGPbqAcqGHsislcqaIXaqmaeqaaOGaeiykaKcabaGaemiDaq3aaSbaaSqaaiabdMgaPbqabaGccqGHsislcqWG0baDdaWgaaWcbaGaemyAaKMaeyOeI0IaeGymaedabeaaaaaaaa@5328@

• SpecificRateraw(ti)=Rateraw(ti)G(ti)
 MathType@MTEF@5@5@+=feaafiart1ev1aaatCvAUfKttLearuWrP9MDH5MBPbIqV92AaeXatLxBI9gBaebbnrfifHhDYfgasaacH8akY=wiFfYdH8Gipec8Eeeu0xXdbba9frFj0=OqFfea0dXdd9vqai=hGuQ8kuc9pgc9s8qqaq=dirpe0xb9q8qiLsFr0=vr0=vr0dc8meaabaqaciaacaGaaeqabaqabeGadaaakeaaieqacqWFtbWucqWFWbaCcqWFLbqzcqWFJbWycqWFPbqAcqWFMbGzcqWFPbqAcqWFJbWycqWFGaaicqWFsbGucqWFHbqycqWF0baDcqWFLbqzdaWgaaWcbaacbmGae4NCaiNae4xyaeMae43DaChabeaakiabcIcaOiabbsha0naaBaaaleaacqqGPbqAaeqaaOGaeiykaKIaeyypa0ZaaSaaaeaacqWFsbGucqWFHbqycqWF0baDcqWFLbqzdaWgaaWcbaGae4NCaiNae4xyaeMae43DaChabeaakiabcIcaOiabdsha0naaBaaaleaacqWGPbqAaeqaaOGaeiykaKcabaGae43raCKaeiikaGIaemiDaq3aaSbaaSqaaiabdMgaPbqabaGccqGGPaqkaaaaaa@5B3F@

• **MSR**_***raw ***_= maximum value of **Specific Rate**_***raw ***_over [0, ***t***_***final***_].

• **TMR**_***raw***_= t_i _where **Rate**_***raw***_(*t*_*i*_) is maximal over [0, ***t***_***final***_].

For the second method, the raw data were first fit to a cubic smoothing spline and the resulting function was transformed to a B-spline (a generalization of the Bézier curve). The spline function was integrated to calculate the AUGC, and it was evaluated at the last time-point to obtain FPI. The specific rate was calculated as the derivative with respect to time, divided by the function (i.e., population growth rate divided by population size), and the MSR was determined from these values. Spot intensities less than 1000 (a conservative threshold for image sensitivity) were not considered in MSR calculation for the spline and raw models (see figure 5).

For growth curve modeling with the logistic equation, the Curve Fitting Toolbox in Matlab was used. Time series data were first filtered to eliminate values that exceeded the initial carrying capacity by more than 10% (see Figs. 5 and 6). An estimate of the initial carrying capacity was determined by first using a smoothing spline to determine the TMR. The spot intensity at TMR was multiplied by 2.2 to estimate the carrying capacity (according to the logistic equation, the population size is at half its carrying capacity at TMR). The TMR spot intensity was scaled by 2.2, instead of 2, to prevent excessive filtering. The following form of the logistic equation was next used to fit the filtered data:

G(t)=K1+e−r(t−l),G(0)<K
 MathType@MTEF@5@5@+=feaafiart1ev1aaatCvAUfKttLearuWrP9MDH5MBPbIqV92AaeXatLxBI9gBaebbnrfifHhDYfgasaacH8akY=wiFfYdH8Gipec8Eeeu0xXdbba9frFj0=OqFfea0dXdd9vqai=hGuQ8kuc9pgc9s8qqaq=dirpe0xb9q8qiLsFr0=vr0=vr0dc8meaabaqaciaacaGaaeqabaqabeGadaaakeaacqWGhbWrcqGGOaakcqWG0baDcqGGPaqkcqGH9aqpdaWcaaqaaiabdUealbqaaiabigdaXiabgUcaRiabdwgaLnaaCaaaleqabaGaeyOeI0IaemOCaiNaeiikaGIaemiDaqNaeyOeI0IaemiBaWMaeiykaKcaaaaakiabcYcaSiabdEeahjabcIcaOiabicdaWiabcMcaPiabgYda8iabdUealbaa@44FC@

The logistic model returns values for the parameters, *K*, *r*, and *l*. *K *is the initial carrying capacity approximating the FPI; *r *is equivalent to the MSR, and *l *is equivalent to TMR.

## Abbreviations

**AUGC**: Area Under Growth Curve.

**CPP**: Cell Proliferation Phenotype

**FPI**: Final Population Intensity

**HTCP**: High Throughput Cellular Phenotyping

**MSR**: Maximum Specific growth Rate.

**PAA**: Phenotypic Array Analysis

**TMR**: Time when Maximum growth Rate is observed

## Authors' contributions

NAS implemented the Java version of YeastXtract, assisted with data collection, image analysis and growth modeling, creation of the figures, and writing the manuscript. RJL and LPZ designed and implemented the YeastXtract image analysis algorithm, building upon work done for SignalViewer [27,28]. BW assisted with image analysis and growth modeling. JLH provided overall direction and was responsible for the experimental design and writing the manuscript.

## Supplementary Material

Additional file 1 Licensing information and user instructions with screenshots of the user interface are described. Click here for file

Additional file 2 **Correlation between image intensities and biomass of spotted cultures**. This file contains the initial dilution of each spot, the spot intensity after 23 hrs, median cell volume, total cell volume, and total cell number measurements for each culture spot, as described in Figure 2. Click here for file

Additional file 3 **Area normalization of spot intensities**. This file contains averaged, normalized and non-normalized spot intensity data from each set of 96 cultures. Arrays made with 2 μL and 4 μL spots from the same liquid culture were compared, as described in Figure 4. Click here for file

Additional file 4 **Cell Proliferation Phenotypes – precision of different growth models**. This file contains the data used to calculate median values and percent standard deviation for Cell Proliferation Phenotypes calculated by different growth models using raw and normalized spot intensity values (See Tables 1 and 2). Click here for file

Additional file 5 **Robustness of growth models against data removal**. Beginning with data in Additional file 4, time points were randomly removed, and MSR was recalculated using each growth model (Fig. 8). This file contains the actual time points and average and standard deviation of MSR of all 96 spots. Click here for file

## Figures and Tables

**Table 1:** Comparison of cell proliferation phenotypes calculated with three different models. Median and percent standard deviation values are shown for four different CPPs calculated by the three growth models tested. Time series spot intensity data from 96 replicate cultures (one cell array) were used [see Additional file 4]. Percent standard deviation is calculated as the standard deviation divided by the median × 100.

	**MSR**		**FPI**		**TMR**		**AUGC**	
**Model**	**Median**	**%Std. Dev.**	**Median**	**%Std. Dev.**	**Median**	**%Std. Dev.**	**Median**	**%Std. Dev.**

**Raw**	.40	23.8	7.96e+4	10.1	29.8	4.4	3.13e+6	9.6
**Spline**	.37	9.1	7.96e+4	10.1	27.3	2.0	3.25e+6	9.5
**Logistic**	.35	4.6	6.7e+4	10.2	26.2	1.2	2.99e+6	9.9

**Table 2:** Effect of spot area normalization on cell proliferation phenotypes. Area-normalized spot intensities were used in place of total intensities to compare the three growth models, as was done in Table 1. Spot area normalization reduced the percent variation for FPI and AUGC, while not affecting MSR or TMR.

	**MSR**		**FPI**		**TMR**		**AUGC**	
**Model**	**Median**	**%Std. Dev.**	**Median**	**%Std. Dev.**	**Median**	**%Std. Dev.**	**Median**	**%Std. Dev.**

**Raw**	.40	23.8	119.1	4.6	29.8	4.4	4724	5.6
**Spline**	.37	9.1	119.1	4.6	27.3	2.0	4886	5.2
**Logistic**	.35	4.1	100.4	6.9	26.2	1.0	4479	6.9
